# An Efficient Homologous Recombination-Based In Situ Protein-Labeling Method in *Verticillium dahliae*

**DOI:** 10.3390/biology13020081

**Published:** 2024-01-28

**Authors:** Jie Yang, Mengran Liu, Yue Jiao, Hui-Shan Guo, Chun-Min Shan, Haiting Wang

**Affiliations:** 1 State Key Laboratory of Plant Genomics, Institute of Microbiology, Chinese Academy of Sciences, Beijing 100101, China; yangjie@im.ac.cn (J.Y.); liumr@im.ac.cn (M.L.); guohs@im.ac.cn (H.-S.G.); 2 University of Chinese Academy of Sciences, Beijing 100049, China; 3 Development Center of Science and Technology, Ministry of Agriculture and Rural Affairs, Beijing 100176, China; jiaoyue@agri.gov.cn

**Keywords:** protein labeling, homologous recombination, *Agrobacterium tumefaciens*-mediated transformation (ATMT), *Verticillium dahliae*, filamentous fungi

## Abstract

**Simple Summary:**

In situ protein labeling through homologous recombination is a powerful method for studying protein function across various fungal organisms. Traditionally, research on gene function in plant pathogens depends on methods like ectopic or overexpression, which may not always reflect natural protein behavior. Our study successfully implemented in situ labeling of proteins in the fungus *Verticillium dahliae*, a plant pathogen, by attaching Flag/GFP tags to proteins’ C-terminus via homologous recombination. We demonstrated the technique’s effectiveness by analyzing the subcellular localization and protein levels, confirming its utility in this organism. This advancement in protein in situ labeling provides a more precise approach to examine protein expression and function in plant pathogens, enhancing our understanding of their biological functions and potentially aiding in the development of disease control strategies.

**Abstract:**

Accurate determination of protein localization, levels, or protein−protein interactions is pivotal for the study of their function, and in situ protein labeling via homologous recombination has emerged as a critical tool in many organisms. While this approach has been refined in various model fungi, the study of protein function in most plant pathogens has predominantly relied on ex situ or overexpression manipulations. To dissect the molecular mechanisms of development and infection for *Verticillium dahliae*, a formidable plant pathogen responsible for vascular wilt diseases, we have established a robust, homologous recombination-based in situ protein labeling strategy in this organism. Utilizing *Agrobacterium tumefaciens*-mediated transformation (ATMT), this methodology facilitates the precise tagging of specific proteins at their C-termini with epitopes, such as GFP and Flag, within the native context of *V. dahliae*. We demonstrate the efficacy of our approach through the in situ labeling of *VdCf2* and *VdDMM2*, followed by subsequent confirmation via subcellular localization and protein-level analyses. Our findings confirm the applicability of homologous recombination for in situ protein labeling in *V. dahliae* and suggest its potential utility across a broad spectrum of filamentous fungi. This labeling method stands to significantly advance the field of functional genomics in plant pathogenic fungi, offering a versatile and powerful tool for the elucidation of protein function.

## 1. Introduction

*Verticillium dahliae,* a soil-borne phytopathogen, is notorious for causing wilt disease across a wide range of plant species by colonizing their vascular systems, predominantly via root infection [1]. This fungus is capable of producing microsclerotia, a type of dormant structure, enabling it to persist in soil for extended periods, even in the absence of a host plant [2,3]. Remarkably, *V. dahliae* can affect over 200 species of dicotyledonous plants, inclusive of vital crops such as cotton, tomato, potato, tobacco, and sunflower, which are all of high economic significance [4,5,6]. Among these, the impact of Verticillium wilt on cotton is particularly devastating, as it is the most destructive disease affecting this crop, causing substantial reductions in both yield and quality [7]. The control of *V. dahliae* presents increasing challenges due to its remarkable environmental adaptability, genetic variability, and intricate pathogenic mechanisms. Despite considerable efforts to elucidate the molecular mechanisms underlying *V. dahliae*’s development and infection processes [8,9,10], many of these aspects remain largely elusive. One possible reason for this challenge may be the limited availability of genetic manipulation tools available for this particular fungus, compared with for other filamentous fungi.

Understanding the molecular basis of growth, development, and infection processes in plant pathogenic fungi, particularly through the functional analysis of key pathogenicity genes, is fundamental for enhancing crop resistance to fungal invasions [11]. Therefore, genome manipulation techniques that facilitate the direct modification or tagging of specific proteins to study their functions within *V. dahliae* are recognized as potent tools for achieving this objective. However, current methods for protein labeling in *V. dahliae* often result in ex situ manipulation or overexpression [8,10], which can lead to several adverse effects. Ectopic accumulation of tagged proteins beyond physiological levels may disrupt normal cellular function, induce negative feedback regulation and may be toxic. Consequently, current protein tagging methods may not accurately represent the native structures or functions of protein complexes, nor true in vivo gene expression profiles in *V. dahliae*.

Homologous recombination offers a precise and efficient method for minimally invasive tagging at endogenous loci, as evidenced in various model organisms including *Saccharomyces cerevisiae* [12,13,14,15,16] and *Drosophila* [17,18,19]. In these organisms endowed with robust homologous recombination systems, precise gene targeting is typically achieved by the integration of a targeting cassette in place of the native genomic sequence via homologous recombination. The targeting cassette commonly incorporates heterologous antibiotic resistance markers which facilitate the selection of cells that have successfully undergone desired genomic integration. This technique facilitates in vivo targeting and manipulation of genes on their normal chromosomal locations and leads to the attachment of tags, such as fluorescent proteins or epitope tags, to these specific proteins. Tagged proteins enable the tracking of protein location within cells or tissues and the monitoring of protein expression and dynamics under native conditions in real-time, aiding in the visualization of their spatial distributions [15,20]. Furthermore, epitope tags (e.g., Flag or HA) can enhance the detection accuracy and purification efficiency of target proteins and diminish costs associated with synthesizing specific antibodies for different proteins [21,22]. In *V. dahliae*, homologous recombination has been widely used for gene knockout, but the currently used protein labeling method often results from ex situ or overexpression manipulation [8,23].

To address challenges associated with elucidating the intrinsic characteristics of pathogenic proteins and to facilitate more accurate biochemical and genetic studies in *V. dahliae*, we focus on the development of an efficient protein in situ tagging method in *V. dahliae*. We harnessed the endogenous homologous recombination machinery of *V. dahliae* to integrate specific tags at precise genomic loci, enabling the in situ C-terminal tagging of proteins of interest. This approach ensures the accurate placement of tags within the genome, thereby optimizing the fidelity of protein tagging.

In situ protein labeling has been extensively employed to study gene function in many model fungi, such as *Neurospora crassa*, *Candida albicans*, and *Fusarium oxysporum.* For instance, in *Candida albicans*, a modular system for PCR-based gene tagging facilitates both C-terminal and N-terminal tagging to study its biological complexity inherent [24,25,26]. *Neurospora crassa* researchers have adopted the double-joint PCR method for protein tagging at native loci, significantly advancing protein localization and functional studies [27]. In *Fusarium oxysporum*, CRISPR/Cas9-mediated endogenous gene tagging has been used to study the expression model of the fungal development-related genes *FoSSO1* and *FoSSO2* [28].

To leverage the homologous recombination-based in situ tagging approach in *V. dahliae,* it is essential to utilize a highly efficient transformation system, which can accurately introduce homologous DNA fragments into the host cells. *Agrobacterium tumefaciens*-mediated transformation (ATMT) is an inherently biological process originally developed for transferring genes in plants. ATMT has been adapted for both stable and transient transformations, offering a cost-effective and straightforward method of preparation and handling [29]. In this method, exogenous protein sequences are inserted into T-DNA (transfer DNA) of the Ti plasmid, which is then transferred into plant cells and subsequently integrated into the plant genome. Beyond its application in plants, ATMT has also been successfully employed to transform yeast, filamentous fungi, and even animal cells [30,31,32,33]. In contrast to the traditional methods that require protoplast preparation for transforming filamentous fungi, ATMT streamlines the process by allowing direct transformation of spores and mycelium. To date, a highly effective ATMT system has been established for gene deletion in *V. dahliae* [23], underscoring the pivotal role of ATMT in gene targeting endeavors across a variety of filamentous fungi [34,35]. ATMT offers several distinct advantages over alternative methods, including the following approaches: (1) high transformation efficiency; (2) the capacity to introduce large DNA fragments with precision; and (3) the introduction of genes at a low copy number. Despite these benefits, ATMT is not without its limitations, which include the following ones: (1) occasional occurrences of multi-copy insertions; (2) a time-intensive transformation process; and (3) an inherently limited host range. To mitigate non-specific insertions during the transformation process, the implementation of the herpes simplex virus thymidine kinase (*HSVtk*) as a negative selection marker has been proposed, which has been shown to enhance the specificity of the transformation [23].

This study centered on two proteins: VdCf2 (*VDAG-08721*)**,** which has been documented to localize to the nucleus [36], and another protein VdDMM2 (*VDAG-00626*), a candidate for nuclear localization. We developed an in situ tagging strategy to append GFP and Flag tags to the C-terminus of VdCf2 and VdDMM2, respectively, within the *V. dahliae* V592 strain. The resultant constructs, VdCf2-GFP, VdCf2-Flag, VdDMM2-GFP, and VdDMM2-Flag, were confirmed for genomic integration via PCR and sequencing. Confocal microscopy facilitated the visualization of the subcellular localization of native GFP-tagged proteins, while the functionality of the Flag-tagged proteins was confirmed through Western blot analysis using anti-Flag antibodies, thus validating the efficacy of the in situ Flag-tagging approach.

## 2. Materials and Methods

### 2.1. Fungal and Bacterial Strains

*Verticillium dahliae* strain V592, provided by Huishan Guo’s Lab (isolated from cotton originating in Xinjiang, China [37]), was the fungal specimen utilized in this study. *Escherichia coli* strain DH5α was employed for plasmid propagation. For fungal transformation, the *Agrobacterium tumefaciens* strain EHA105 was used. Transformants were incubated and selected on Potato Dextrose Agar (PDA) medium, supplemented with 5-fluoro-2′deoxyuridine (5FU) at a concentration of 20 µg/L and hygromycin B at 50 µg/mL. Competent cells of *Escherichia coli* DH5α and *Agrobacterium tumefaciens* EHA105 were sourced from Beijing Tsingke Biotech Co., Ltd., Beijing, China.

### 2.2. Vectors

The foundational vector, pGKO-HPT, provided by Huishan Guo’s Lab containing the herpes simplex virus thymidine kinase (*HSVtk*) negative selection marker, to inhibit ectopic integration and the hygromycin B resistance gene. GFP/Flag cassettes were PCR-amplified from pHM1-GFP and pFA6a-3flag vector, respectively, and then cloned into the StuI-digested pGKO-HPT vector via homologous recombination, to generate the pGKO-tag-HPT vector for in situ tagging.

### 2.3. Construction and Transformation

In situ tagging vectors were constructed by PCR-amplifying 1 kb upstream (5’) and downstream (3’) flanking sequences adjacent to the target protein’s stop codon, excluding the stop codon itself. These sequences were then inserted into the PacI restriction cleavage sites flanking the tagging box (Flag or GFP) and the hygromycin resistance cassette within the pGKO-tag-HPT vector via homologous recombination. The completed in situ tagging vectors were then introduced into *Agrobacterium* strain EHA105 which was subsequently co-cultivated with the *V. dahliae* V592 strain. Transformants were selected on potato dextrose agar (PDA) plates supplemented with 5-fluoro-2′deoxyuridine (5FU) and hygromycin. Ultimately, the primer pairs V1/Hpt-R and V2/Hpt-F were used to PCR, confirming the successful integration of tags for target proteins in *V. dahliae* transformants.

### 2.4. Confocal Laser Scanning Microscopy (CLSM)

*V. dahliae* conidia grown on Potato Dextrose Agar (PDA) medium were harvested and rinsed once with double distilled water (ddH_2_O), to remove any residual medium. A suitable volume of the conidial solution was placed on a silicified glass slide, air-dried and then sealed with a DAPI dye solution. After a 10 min incubation, samples were examined under a Leica TCS SP8 confocal microscope. GFP fluorescence was detected at an excitation wavelength of 488 nm, while DPAI-stained nuclei were visualized at 350 nm.

### 2.5. Protein Detection via Western Blotting

Spores of *V. dahliae* were harvested and washed with PBS and then resuspended in 3 mL of lysis buffer (50 mM Tris-HCl pH 7.4, 150 mM NaCl, 1 mM EDTA, 1% Triton, and 1 × protease inhibitor cocktail). The cell suspension jelly was fast-frozen in liquid nitrogen and pulverized with a grinder (Xianfeng Scientific Instrument, Wuhan, China, 70 Hz, 1.5 min, 3 times). The lysate was then diluted to 10 mL with lysis buffer and incubated with 150 μL Flag M2 agarose slurry (Sigma, Burlington, MA, USA, A2220) at 4 °C overnight. The resin was washed three times with 1 mL TBS buffer each, and the tagged proteins were eluted with 100 μL 0.1 M glycine-HCl (pH 2.5) for 5–10 min. The eluate was neutralized with 7.2 μL Tris-HCl (pH 8.0). Detection of VdCf2-Flag and VdDMM2-Flag was performed using Western blot analysis with anti-Flag antibodies (Sigma, F3165).

All primers used are listed in Supplementary Table S1.

## 3. Results

### 3.1. The General Strategy for In Situ C-Terminus Tagging in V. dahliae

**Figure 1:**
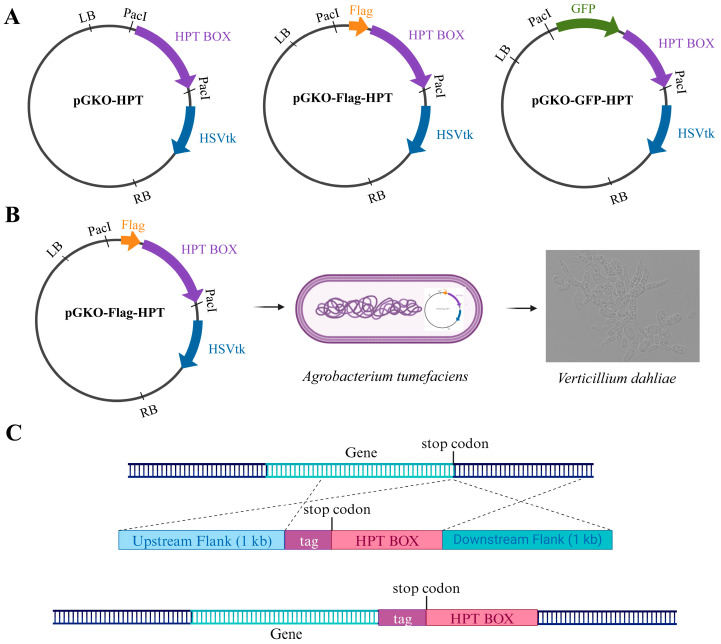
Overview of the in situ protein labeling methodology. (**A**) Construction of the tagging vectors utilized in this study. (**B**) Stepwise protocol for *Agrobacterium tumefaciens*-mediated transformation (ATMT). (**C**) Schematic of the design strategy for in situ tagging and the corresponding genomic integration resulting from the process. HPT BOX—hygromycin B resistance gene; *HSVtk*—herpes simplex virus thymidine kinase.

To develop an in situ tagging system for *V. dahliae* via ATMT, our initial step was to engineer appropriate vectors. The foundational vector, pGKO-HPT, was equipped with a herpes simplex virus thymidine kinase (*HSVtk*) negative-selection marker to curtail ectopic integration and confer hygromycin B resistance for selection. We modified this vector by inserting the desired tag upstream of the HPT gene via homologous recombination, thus creating the pGKO-tag-HPT vector for protein tagging. Using this strategy, we synthesized the pGKO-Flag-HPT and pGKO-GFP-HPT vectors, containing the Flag and GFP tags, respectively (Figure 1A).

To generate the in situ tagging vector for a specific protein, we PCR-amplified the 1 kb upstream (5’) and downstream (3’) flanking sequences adjacent to the target protein’s stop codon, excluding the stop codon itself, for insertion into the PacI restriction cutting sites flanking the tagging box (Flag or GFP) and the hygromycin resistance cassette within the pGKO-tag-HPT vector. To ensure proper orientation, the primers were designed to include the flanking 23 bp homology sequence, with the PacI site sequence for both sides. The assembled in situ tagging vectors were then introduced into *Agrobacterium* strain EHA105. Following co-cultivation with the V592 strain cells of *V. dahliae*, the positive transformants were selected on potato dextrose agar (PDA) plates supplemented with 5-fluoro-2′deoxyuridine (5FU) and hygromycin B (Figure 1B). The ultimate result is the in situ integration of the tag and the hygromycin-resistant cassette at the C-terminus of the target protein, mediated by *V. dahliae*’s intrinsic homologous recombination system (Figure 1C).

### 3.2. In Situ GFP Tagging of VdCf2 and VdDMM2 Using the pGKO-GFP-HPT Vector

**Figure 2:**
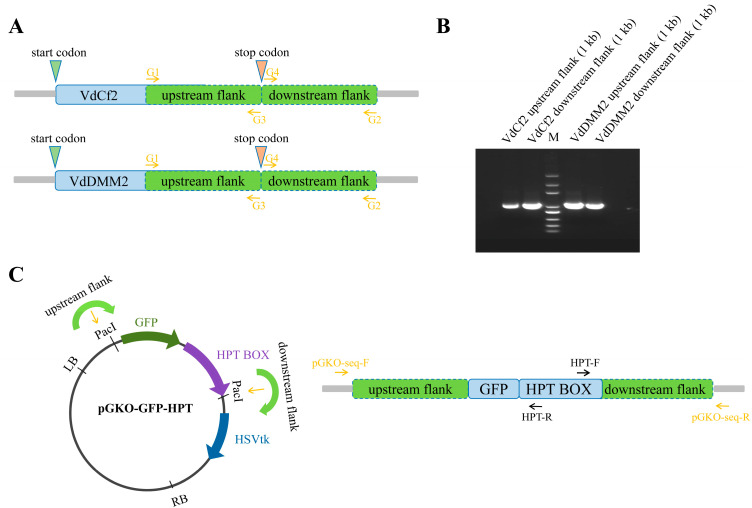
Construction of in situ GFP tagging vectors for *VdCf2* and *VdDMM2*. (**A**) Schematic of primer design specific to the in situ GFP tagging approach. (**B**) Agarose gel electrophoresis confirmation of the PCR-amplified upstream and downstream flanking regions of insertions at *VdCf2* and *VdDMM2* genes loci. (**C**) Schematic of the in situ GFP tagging vector construction, including the primer sets within the HPT BOX, employed for validating the integration of the upstream and downstream flanking sequences into the pGKO-GFP-HPT vector.

To assess the capability of the pGKO-GFP-HPT vector in mediating in situ GFP tagging of specific proteins, we engineered the in situ tagging constructs for *VdCf2* and *VdDMM2*, incorporating the flanking sequences both upstream and downstream of the respective genes. Using *VdCf2* as a representative example, we commenced by synthesizing primer pairs VdCf2-G1/VdCf2-G3 and VdCf2-G4/VdCf2-G2 (Table S1) to PCR amplify the upstream and downstream flanking regions, respectively, based on the reference genome sequence of *V. dahliae* strain *VdLs.17* (Figure 2A) [38]. The pGKO-GFP-HPT vector was subjected to PacI digestion to liberate the vector backbone along with the GFP-HPT BOX. Subsequent to this step, the amplified flanking sequences (Figure 2B) were seamlessly integrated into the PacI sites through homologous recombination, generating the pGKO-VdCf2-GFP-HPT construct (Figure 2C). Verification of the construct was meticulously performed using PCR with the primer pairs pGKO-seq-F/Hpt-R and Hpt-F/pGKO-seq-R (Figure 2C and Table S1). Upon confirmation, the resultant in situ tagging vectors were finally introduced into *V. dahliae* via ATMT, as described above. The methodology adopted for the construction of the *VdDMM2* in situ tagging vector mirrored that of *VdCf2*, ensuring consistency in our approach.

### 3.3. Molecular Confirmation of C-Terminal GFP Tagging for VdCf2 and VdDMM2 in V. dahliae

**Figure 3:**
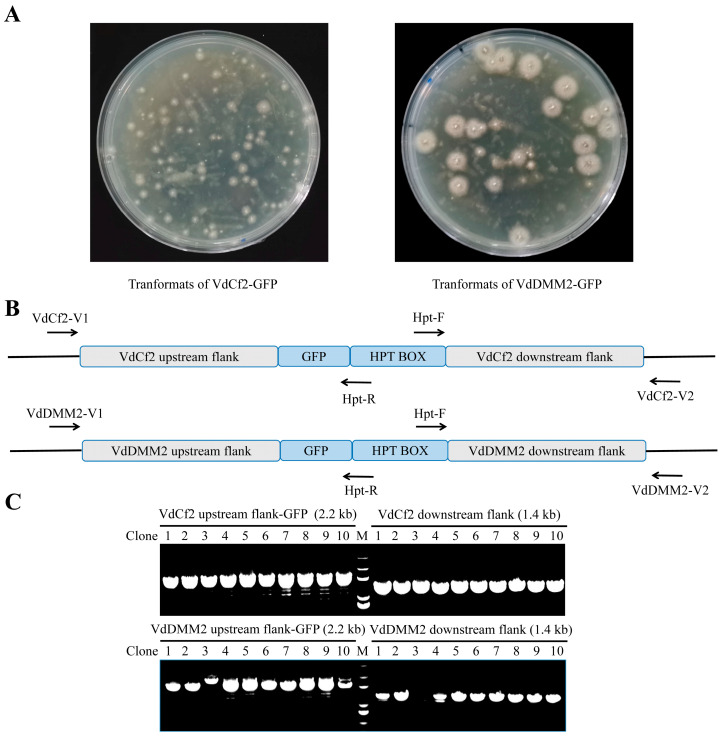
The verification of in situ GFP tagging for VdCf2 and VdDMM2 in *V. dahliae*. (**A**) Transformants of VdCf2-GFP and VdDMM2-GFP grown on potato dextrose agar (PDA) plates with 5-fluoro-2′deoxyuridine (5FU) and hygromycin B. (**B**) Schematic of verification primer pairs specifically designed for the assessment of successful tagging. (**C**) PCR validation results for demonstrating the successful in situ GFP tagging of VdCf2 and VdDMM2, utilizing primer pairs VdCf2-V1/Hpt-R, VdCf2-V2/Hpt-F and VdDMM2-V1/Hpt-R, VdDMM2-V2/Hpt-F.

To confirm the successful in situ GFP tagging of *VdCf2* transformants, we isolated the genomic DNA from the candidate transformants (Figure 3A) and then designed the verification primer pair, to bind approximately 200 bp upstream of the upstream flanking sequence (referred to as VdCf2-V1) and 200 bp downstream of the downstream flanking sequence (referred to as VdCf2-V2), respectively.The primer pair VdCf2-V1/Hpt-R was utilized to ascertain the insert of the correct upstream integration site, while the VdCf2-V2/Hpt-F primer pair was applied for downstream validation (Figure 3B and Table S1). The methodology adopted for the verification of the *VdDMM2* in situ GFP tagging was similar to that for the verification of *VdCf2*. Our PCR verification results demonstrated a remarkably high efficiency of in situ GFP tagging for *VdCf2* and *VdDMM2*, with success rates of 100% and 90%, respectively (Figure 3C). To further validate the fidelity, sequencing of PCR products from the positive transformants was conducted, confirming the accurate integration of the in situ GFP tags within the *VdCf2* and *VdDMM2* genes.

### 3.4. Subcellular Localization for In Situ Tagged Endogenous VdCf2-GFP and VdDMM2-GFP in V. dahliae

**Figure 4:**
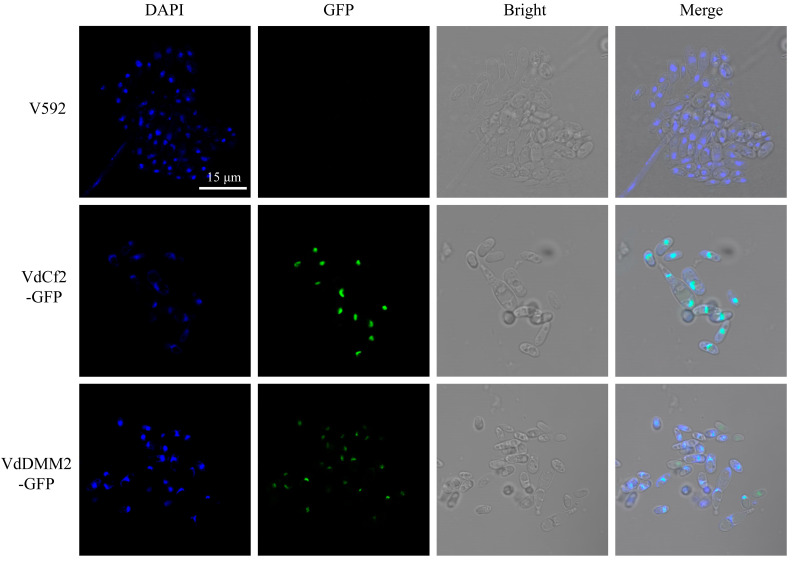
The subcellular localization for in situ GFP-tagged *VdCf2* and *VdDMM2* in *V. dahliae* cells. The fluorescence imaging of VdCf2-GFP and VdDMM2-GFP under confocal microscope (Leica TCS SP8). Scale bar: 15 μm.

To explore the subcellular localization of VdCf2-GFP and VdDMM2-GFP in *V. dahliae*, we observed the in situ tagged VdCf2-GFP and VdDMM2-GFP proteins within *V. dahliae* cells using a confocal microscope (Leica TCS SP8). Previous studies have indicated nuclear localization for both proteins. Hence, we employed 4’,6-diamidino-2-phenylindole (DAPI) staining to facilitate the identification of the nuclei in the cells. Confocal microscopy revealed distinct bright green foci within the positive transformants (Figure 4), indicative of the successful GFP-tagging of the VdCf2 and VdDMM2 proteins. Notably, colocalization of the GFP fluorescence with the DAPI-stained nuclei was observed (Figure 4), conclusively demonstrating that the endogenous VdCf2-GFP and VdDMM2-GFP fusion proteins indeed localized to the nucleus.

### 3.5. Assessment of Versatility for in In Situ Tagging System within V. dahliae

**Figure 5:**
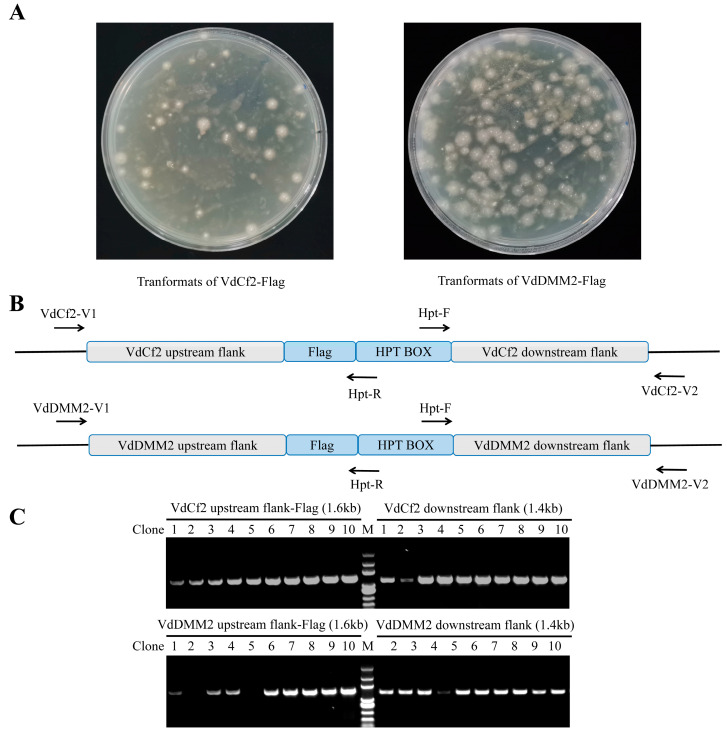
The verification of in situ flag tagging for *VdCf2* and *VdDMM2* in *V. dahliae*. (**A**) Transformants of VdCf2-Flag and VdDMM2-Flag grown on PDA plates with 5FU and hygromycin B. (**B**) Schematic of verification primer pairs specifically designed for the assessment of successful tagging. (**C**) PCR validation results for demonstrating the successful in situ Flag tagging of *VdCf2* and *VdDMM2*, utilizing primer pairs VdCf2-V1/Hpt-R, VdCf2-V2/Hpt-F, VdDMM2-V1/Hpt-R, and VdDMM2-V2/Hpt-F.

To evaluate the broad applicability of our in situ tagging strategy for the incorporation of various epitope tags into *V. dahliae* proteins, we extended our approach to include the Flag epitope. Utilizing the pGKO-Flag-HPT vector, we performed in situ *Flag* tagging for the *VdCf2* and *VdDMM2* genes, mirroring the methodology employed for GFP tagging. The procedure entailed the integration of upstream and downstream flanking sequences adjacent to the Flag tag and HPT selection marker, followed by transformation into *V. dahliae.* To molecularly verify the transformants (Figure 5A), we also designed the verification primers VdCf2-V1/V2 and VdDMM2-V1/V2, which were paired with Hpt-R and Hpt-F, respectively, for PCR (Figure 5B and Table S1). In addition, the products were then sequenced for further verification. The efficiency of generating VdCf2-Flag and VdDMM2-Flag tagged strains was still impressively high, with success rates of 100% and 80%, respectively (Figure 5C).

### 3.6. Validation of Endogenous VdCf2-Flag and VdDMM2-Flag Fusion Proteins in V. dahliae by Western Blot Analysis

**Figure 6:**
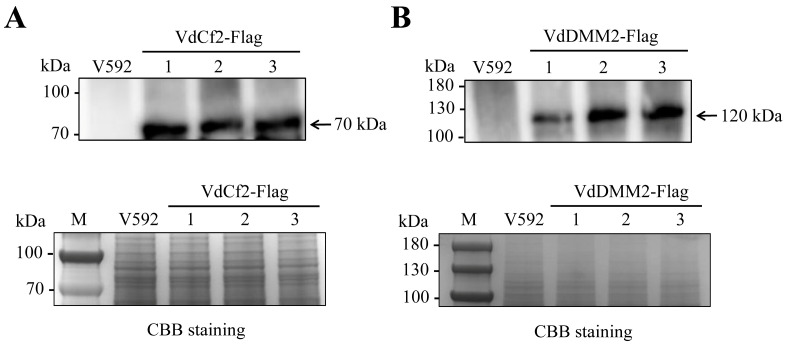
Western blot analysis of *VdCf2* and *VdDMM2* in situ Flag tagging in *V. dahliae*. (**A**) Western blot confirmation of VdCf2-Flag protein expression, with total protein loading visualized by Coomassie brilliant blue (CBB) staining. (**B**) Western blot confirmation of VdDMM2-Flag protein expression, with total protein loading visualized by CBB staining.

To confirm the in vivo expression of Flag-tagged VdCf2 and VdDMM2 fusion proteins in *V. dahliae*, we isolated proteins from positive transformants expressing VdCf2-Flag and VdDMM2-Flag, using anti-Flag agarose beads (Sigma, A2220). Subsequent Western blot analysis was conducted utilizing an anti-Flag antibody (Sigma, F3165), to detect the presence of the fusion proteins. The resulting immunoblots revealed distinct bands corresponding to VdCf2-Flag and VdDMM2-Flag, thereby verifying their successful expression in the transformants. In contrast, no such bands were detected in the wild-type strain, confirming the specificity of the Flag-tagging and the absence of endogenous Flag-tagged proteins (Figure 6 and Figure S1).

## 4. Discussion

The soilborne fungal pathogen *V. dahliae* presents a significant challenge to agriculture due to its high virulence, broad host range, and the difficulty associated with its management. In recent years, host-induced gene silencing (HIGS), a technique derived from trans-kingdom RNA interference (RNAi), has emerged as an innovative strategy for the mitigation of *V. dahliae* infections [39]. This approach hinges on the identification and utilization of fungal genes that are pivotal for its growth and pathogenicity, underscoring the importance of functional genomics in controlling of this pathogen.

Protein tagging is a fundamental technique for unraveling gene function and localization. Within fungal research, ectopic expression has been proven an invaluable tool. Notably, in *Saccharomyces cerevisiae*, the Yeast two-hybrid system has been extensively employed for ectopic protein tagging, shedding light on intricate protein interactions. Subsequent advances include the epitope tagging of each protein’s open reading frame (ORF), facilitating the construction of a comprehensive library of hybrid proteins, to dissect protein−protein interactions [40]. While gene localization studies have predominantly relied on overexpression systems to yield more robust signals [8,9], the use of ectopic or overexpression tags is not without its detriments. Such approaches can inadvertently alter the native expression levels and functionality of the protein, disrupt complex assembly, lead to gene silencing or even result in lethality of the cells. Therefore, the pursuit of more refined gene tagging methods that preserve the endogenous context of target genes remains a critical objective for the accurate interpretation of protein function and interaction networks within *V. dahliae*.

Currently, a suite of gene in situ tagging methodologies has been developed and refined, including homologous recombination [41], and CRISPR/Cas9-mediated endogenous gene tagging [28], each contributing valuable insights into fungal studies. In contrast to ectopic or overexpression of proteins, in situ tagging offers a methodological advantage by maintaining the native levels of endogenous proteins. This method has been successfully implemented in various model fungal organisms, including *Saccharomyces cerevisiae* and *Schizosaccharomyces pombe* [12,15,21,22], setting a precedent for its application in fungal studies. Despite its proven efficacy in model systems, the adoption of an in situ tagging system in plant pathogenic fungi, particularly in *V. dahliae*, remains scant. This situation significantly constrains our capacity to study the structural, functional, and physiological aspects of this pathogen. The integration of in situ tagging methods into the research toolkit for these filamentous pathogen fungi represents a formidable approach to elucidate the localization, interactions, and dynamic behavior of intracellular molecules. Its application is poised to yield new insights into the cellular biology of *V. dahliae*, thereby advancing our understanding of its pathogenic mechanisms and informing the development of novel control strategies.

In this study, we validated the effectiveness of the homologous recombination-based in situ tagging method in the filamentous pathogen fungus *V. dahliae* V592, exemplified by the successful tagging of *VdCf2* and *VdDMM2* with Flag and GFP, respectively. After in situ tagging, the resultant fusion proteins were expressed at normal levels within *V. dahliae*, and their intracellular localization was consistent with previously reported observations. The application of Flag tagging, possibly followed by affinity purification and mass spectrometry analysis, has set the stage for probing the interacting partners of these proteins, thereby facilitating a deeper understanding of protein complexes, which is a pivotal aspect of functional proteomics. Moreover, this methodology is not confined to Flag or GFP tags. It also enables the integration of alternative tags or inducible promoters into *V. dahliae* via homologous recombination. This advancement is particularly useful, as it provides a strategy to surmount the inherent autofluorescence often encountered in fungal cells, and it paves the way for the regulable expression of target proteins.

The method described, while effective for protein C-terminal labeling, is still not without its limitations. For example, the signal of fusion protein may be indetectable, if the C-terminal is internally folded within the secondary structure. Furthermore, post-translational cleavage at the tag fusion site may lead to the detachment of the tag from the target protein. While in situ labeling of protein C-termini preserves the natural expression levels within cells, it carries the potential risk of altering the structural stability of proteins. This can result in protein degradation or impact the catalytic activity, potentially inducing phenotypic changes similar to the gene knockout mutants.

To address these issues, sometimes N-terminal labeling is necessary, and the gene promoter region can be utilized as the upstream homologous arm. The decision to tag the C-terminal or N-terminal end of a target protein largely hinges on the protein’s characteristics, its folding properties, and whether the chosen terminus carries functional significance. Each protein’s unique attributes must be carefully considered to ensure that tagging does not interfere with its native function or stability, thereby allowing for accurate functional analyses.

Looking ahead, future work should also concentrate on enhancing the efficiency of transformation techniques, given the intricacy and laborious nature of ATMT for *V. dahliae*. Additionally, the expansion of the tagging types is imperative to fully harness the capabilities of this method and to propel the functional genomics of *V. dahliae* or other plant pathogens into new frontiers.

## 5. Conclusions

In summary, limited research on the soil-borne fungal pathogen *Verticillium dahliae’s* genes related to growth, development, and pathogenic mechanisms stems from a lack of genetic manipulation methods. Protein labeling emerges as a crucial tool for exploring gene function and localization. Our study introduces protein in situ labeling techniques in *Verticillium dahliae*, emphasizing natural protein expression and function over ectopic or overexpressed proteins. Integrating these methods into the toolkit for studying plant pathogenic filamentous fungi provides a potent method to investigate molecular localization, interactions, and dynamic behavior within fungal cells. This advancement in protein in situ tagging technology is poised to drive the exploration of *Verticillium dahliae’s* gene function, deepening our understanding of pathogenic mechanisms and aiding the development of innovative control strategies.

## Data Availability

Data are contained within the article and Supplementary Materials.

## Supplementary Materials

The following supporting information can be downloaded at: https://www.mdpi.com/article/10.3390/biology13020081/s1, Figure S1: Original images of Figure 6; Table S1: Primers used in this study.
