# Supplementary material for: An Efficient Homologous Recombination-Based In Situ Protein-Labeling Method in Verticillium dahliae
Source: Biology (Basel). 2024 Jan 28;13(2):81. doi: 10.3390/biology13020081 (PMC10886240; doi:10.3390/biology13020081)
Supplement: Supplementary file 1 [file biology-13-00081-s001.zip › biology-2804735-supplementary.pdf]

Supplementary Table S1. Primers used in this study.

Gene	Primers	Sequences (5′→3′)	Application
VdCf2	G1	AATTCGAGCTCGCTGAGGGTTTAATTA<br>AGATGCTCTCGCGCGACAC	*In situ* GFP and Flag tagging of *VdCf2*
	G3	TCGATGGGCCCGCTGAGGACTTAATT<br>AATGCCTTGGGCGTGAGGGC	
	G4	CCCCGACTAGTGCTGAGGCATTAATT<br>AAATGATGCGTCAAGTCCAAT	
	G2	TCACGAAGCTTGCTGAGGTCTTAATTA<br>ATGGTCGCACGAAATGGAG	
	V1	GCACAAGTGCCCGTACTG	
	V2	GTTCCCACATCCTGGCGC	
VdDMM2	G1	AATTCGAGCTCGCTGAGGGTTTAATTA<br>ACCAAGCGGCGCCGGACGC	*In situ* GFP and Flag tagging of *VdDMM2*
	G3	TCGATGGGCCCGCTGAGGACTTAATT<br>AACCGCGAATTCATGCCCGC	
	G4	CCCCGACTAGTGCTGAGGCATTAATT<br>AAAGAATGTTCGGACCCGGT	
	G2	TCACGAAGCTTGCTGAGGTCTTAATTA<br>ATAGGAACAGAAACCTTGC	
	V1	AGATTACTTCAACACGGCTC	
	V2	CTCCCGGAACCATCATCTTCC	
	Hpt-F	TCTCCTTGCATGCACCATTCCTTG	Detection of VdCf2 - GFP / Flag and VdDMM2- GFP / Flag
	HPT-R	GCAGCTATTTACCCGCAGGA	
	PGKO-seq-F	CCAATTACACCCTTTGCGCC	
	PGKO-seq-R	AAGAAAACCCACGCCACCTA	

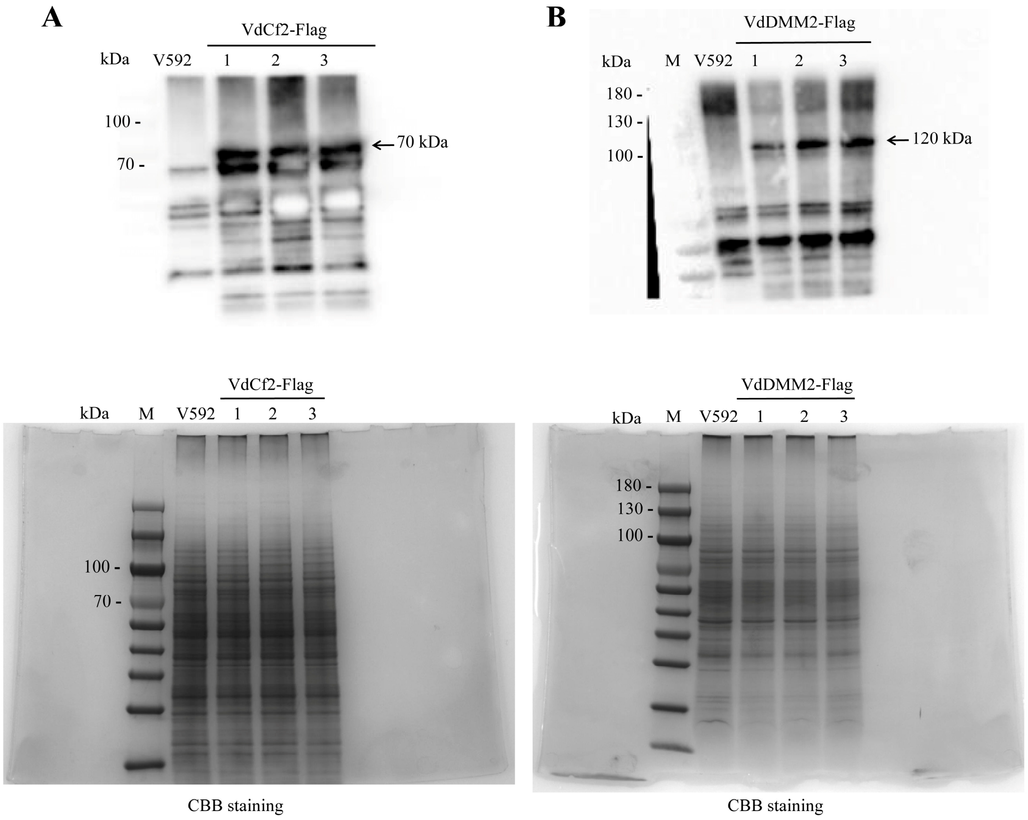

Supplementary Figure S1. Original Images of Western Blot Analysis of *VdCf2* and *VdDMM2 in situ* Flag tagging in *V. dahliae*. (A)Western blot confirmation of VdCf2-Flag protein expression, with total protein loading visualized by Coomassie brilliant blue (CBB) staining. (B) Western blot confirmation of VdDMM2-Flag protein expression, with total protein loading visualized by CBB staining.
